# Early Diagnosis and Monitoring of Neurodegenerative Langerhans Cell Histiocytosis

**DOI:** 10.1371/journal.pone.0131635

**Published:** 2015-07-15

**Authors:** Elena Sieni, Carmen Barba, Marzia Mortilla, Sara Savelli, Laura Grisotto, Gianpiero Di Giacomo, Katiuscia Romano, Claudio Fonda, Annibale Biggeri, Renzo Guerrini, Maurizio Aricò

**Affiliations:** 1 Pediatric Hematology Oncology, Meyer Children's University Hospital, Florence, Italy; 2 Pediatric Neurology, Meyer Children's University Hospital, Florence, Italy; 3 Pediatric Radiology, Meyer Children's University Hospital, Florence, Italy; 4 Department of Diagnostic Imaging, IRCCS Bambino Gesù Children's Hospital, Rome, Italy; 5 Department of Statistics, Informatics and Applications “G. Parenti”, University of Florence, Florence, Italy; 6 Biostatistics Unit, ISPO Cancer Prevention and Research Institute, Florence, Italy; 7 Direzione Generale, Azienda Sanitaria Provinciale 7, Ragusa, Italy; University of Manchester, UNITED KINGDOM

## Abstract

**Background:**

Neurodegenerative Langerhans Cell Histiocytosis (ND-LCH) is a rare, unpredictable consequence that may devastate the quality of life of patients cured from LCH. We prospectively applied a multidisciplinary diagnostic work-up to early identify and follow-up patients with ND-LCH, with the ultimate goal of better determining the appropriate time for starting therapy.

**Methods:**

We studied 27 children and young adults with either ND-LCH verified by structural magnetic resonance imaging (MRI) (group 1) or specific risk factors for (diabetes insipidus, craniofacial bone lesions), but no evidence of, neurodegenerative MRI changes (group 2). All patients underwent clinical, neurophysiological and MRI studies.

**Results:**

Seventeen patients had MRI alterations typical for ND-LCH. Nine showed neurological impairment but only three were symptomatic; 11 had abnormal somatosensory evoked potentials (SEPs), and five had abnormal brainstem auditory evoked potentials (BAEPs). MR spectroscopy (MRS) showed reduced cerebellar NAA/Cr ratio in nine patients. SEPs showed sensitivity, specificity, positive predictive value (PPV) and negative predictive value (NPV) for predicting ND-LCH of 70.6% (95%CI, 44.0%-89.7%), 100% (69.2%-100%), 100% (73.5%-100%), and 66.7% (38.4%-88.2%), respectively. Repeated investigations in group 1 revealed increasingly abnormal EP parameters, or neurological examination, or both, in nine of fifteen patients while MRI remained unchanged in all but one patient.

**Conclusion:**

A targeted MRI study should be performed in all patients with risk factors for ND-LCH for early identification of demyelination. The combined use of SEPs and careful neurological evaluation may represent a valuable, low-cost, well-tolerated and easily available methodology to monitor patients from pre-symptomatic to symptomatic stages. We suggest a multidisciplinary protocol including clinical, MRS, and neurophysiological investigations to identify a population target for future therapeutic trials.

## Introduction

Langerhans cell histiocytosis (LCH) is a rare disease of the dendritic cell system that may occur at any age, with a peak incidence between 5 and 10 years[1]. Its annual incidence has been estimated to be one case in 200.000 subjects at risk in two independent studies in France[2] and in Japan[3]. Its histopathologic landmark is a granulomatous infiltrate of histiocytes of LC phenotype (CD1a+), interspersed with varying proportions of macrophages, T-lymphocytes, eosinophils and multinucleated giant cells. The mechanisms underlying LCH have not been fully clarified. The demonstration that lesional Langerhans cells are clonal, along with the recent discovery of activating BRAF mutations in LCH cells, has been taken as a strong support to the concept that LCH is a neoplastic disease[4, 5]. Clinical presentation and course of LCH are widely heterogeneous and may range from self-healing isolated skin or bone lesion(s), to disseminated disease with potentially lethal tissue damage[1]. Survivors may experience permanent consequences, which affect the CNS in up to 30% of cases[6, 7].

Different MRI patterns of CNS involvement have been described[8, 9, 10]. The most common is the hypothalamic-pituitary granuloma, with diabetes insipidus (DI) and anterior pituitary dysfunction. Less frequently granulomatous lesions have been detected in the meninges, the choroid plexus, the pineal gland or the cerebral parenchyma. The second most frequent MRI pattern is the “neurodegenerative LCH” (ND-LCH), characterized by bilateral symmetric alterations of variable signal intensity located in the cerebellar grey matter, sometimes extending to the underlying white matter, in the basal ganglia and brainstem. Histopathology from cerebellar biopsies and autopsies revealed neuronal loss, axonal degeneration and a profound T-cell inflammation[11].

ND-LCH is a potentially devastating, permanent consequence of LCH. Its real prevalence is still unclear, ranging between 1% and 25% in different cohorts [12–17]. Patients with “*cranio-facial*” lesions involving the orbital, temporal, sphenoid, ethmoid or mastoid bones, the paranasal sinuses, the anterior or middle cranial fossa and/or with DI, carry a higher risk of developing this serious complication[7–18].The pathogenic process might propagate from long-standing granulomatous lesions of the craniofacial bones to the intracranial space, and then stimulate a chemokine/cytokine tissue damage or initiate an autoimmune response to brain components[11].

Despite a relatively homogeneous MRI pattern, the clinical manifestations of ND-LCH are extremely heterogeneous, ranging from minimal or no neurological impairment, to a severe clinical picture including ataxia, spastic quadriparesis, intellectual disability and psychiatric symptoms[9, 12–16]. Although the likelihood of observing clinical manifestations appears to be related to the observation time[12], some patients unexpectedly remain asymptomatic for years despite exhibiting MRI abnormalities[9, 12–17, 19]. Previous attempts to characterize the clinical and functional pattern of ND-LCH and the progression of this complication have yielded inconsistent results[15, 19]. Small retrospective studies described global cognitive impairment with mild memory, attention, and perceptual-organizational changes[13, 19–21]. Abnormal visual (VEPs) and brainstem evoked potentials (BAEPs), and EEG have been reported in a few patients with ND-LCH[15, 19]. Somatosensory evoked potentials (SEPs) have been used for characterizing cerebellar and brainstem dysfunction in several neurological diseases [22–24] but their diagnostic yield has not been assessedin ND-LCH.Thus, there is no evidence supporting the usefulness of neurophysiological examinations in patients with ND-LCH. A standardized diagnostic approach able to predict the clinical progression in patients with ND-LCH might represent a response to an unmet medical need.

Another largely unmet need concerns effective ND-LCH treatment. Various approaches, including chemotherapy, anti-inflammatory, anti-angiogenic and immune-modulatory medications, have been used in a few symptomatic patients yielding deceptive results. Based on these data from sporadic reports, the current prospective therapeutic trial LCH-IV of the Histiocyte Society (EudraCT number: 2011-001699-20) suggest either intravenous immune globulin or cytarabine in patients with clinical signs of ND-LCH.

In this study we describe clinical features, MRI and neurophysiological alterations in ND-LCH and propose a diagnostic work-up for early identification and follow-up of ND-LCH, with the ultimate goal of selecting patients for a future therapeutic intervention in pre- or pauci-symptomatic stages.

## Methods

### Primary and secondary endpoints

Our primary endpoints were: a) to analyze clinical features, MRI and neurophysiological alterations in ND-LCH and b) propose a diagnostic work-up for early identification and follow-up of ND-LCH. Secondary endpoints were: a) to follow-up a group of patients with ND-LCH and b) to define selection criteria for a future therapeutic intervention in pre- or pauci-symptomatic stages.

### Study population and diagnostic protocol

As the national referral center for LCH, we launched a survey among all the Italian pediatric hematology-oncology centers to evaluate patients with LCH, either newly diagnosed, on therapy or in follow-up, using a new integrated diagnostic protocol for ND-LCH. In our study we defined as affected by ND-LCH all patients showing typical MRI alterations and we considered structural MRI evaluation as the reference standard[9, 11, 16]. Inclusion criteria were: informed consent, histologically proven LCH, and presence of risk factors for ND-LCH. Exclusion criteria were: presence of non-LCH related MRI alterations and incomplete MRI protocol. Risk factors were defined as cranio-facial bone lesions and/or DI at the study entry[18].

The diagnostic protocol included the following steps: neurological examination (NE), neuropsychological and neurophysiological study (SEPs, BAEPs, VEPs, EEG), MRspectroscopy (MRS) (see S1 Methods for details). The neuroradiologists who evaluated structural MRI were blinded to the results of the diagnostic protocol and the physicist who analysed MRS was blinded to the results of. neuropsychological,neurophysiological, and structural MRI studies. Neuroradiologists were aware of the results of previous MRI investigations, if any.

Written informed consent was obtained from the parents of affected children or directly from the patients if they were older than 18 years of age.

### Ethics Statement

The study was approved by the Meyer Children’s University Hospital Ethics Committee in Florence, Italy and has been performed in accordance with the ethical standards laid down in the 1964 Declaration of Helsinki and its later amendments.

### Data collection and statistical analysis

We stored demographics and data on organ involvement, treatment, and disease course in a dedicated database. On the basis of the reference standard findings, we classified patients in two groups: group 1 including patients with ND-LCH and group 2 including patients without ND-LCH, but at risk to develop it.

The date of onset of LCH was that of either histological diagnosis or onset of isolated DI. We summarized continuous time variables (age at LCH onset, age at the study entry) by median and range, and made comparisons between groups using Mann-Whitney test. When appropriate, confidence intervals (CI) were calculated using exact likelihood. We set level of significance at 5% two sided.

We analyzed diagnostic accuracy by calculating sensitivity, specificity, Receiver operating characteristic (ROC) curves, positive and negative predictive values for all the diagnostic tests included in the protocol.

Considering as outcome the ordinal MRI grading, we evaluated the discrimination performance of the diagnostic tests using Harrell’s c, a generalization of the area under the ROC curve[25, 26].

To evaluate the improvement in discrimination of adding to the best performing diagnostics a further test, we calculated the Integrated Discrimination Improvement (IDI) statistics[25]. We also reported the components of IDI, i.e. the average improvement in sensitivity for each level of grading MRI. We conducted all statistical analyses using STATA 12 (T Stat s.r.l.).

## Results

### Study population (S1 Table, Table 1)

Between April 2010 and February 2012, we enrolled 30 consecutive patients. We subsequentely excluded three patients because they either did not complete the MRI protocol (n = 2) or showed brainstem abnormalities of uncertain nature at MRI (n = 1).

**Table 1:** Main characteristics of the 27 patients with LCH divided in two groups according to the presence of either MRI alterations specific for ND-LCH (Group 1) or only of risk factors for ND-LCH (Group 2).

	Group 1N = 17	Group 2N = 10	Difference(95% confidence interval)p-value
*Gender*	9 M, 8 F	7 M, 3 F	
*Median age at study entry (range)*	8.2 years(1.7 to 27.5 years;quartiles: 5, 8.2, 11.7 years)	10.2 years(3.7 to 16.4 yearsquartiles: 5.8, 10.2, 14.8 years)	-2.02 years(-8.97;4.6)p = 0.688
*Median time between the onset of LCH and the first MRI evaluation*	4.2 years	2.4 years	1.8 years(-1.3;5.1)p = 0.248
*Median time between the l onset of LCH and the first observation of ND-LCH at MRI*	4.2 years(quartiles: 0.8; 2.5; 4.2; 6.5; 14.5 years).	n.a.	
*Median age at onset s of LCH*	2 years(range, 4 months -18 years;quartiles: 0.9, 2 and 3.3 years)	5.5 years(range, 18 months -15 years; quartiles: 3.2, 5.5 and 9.5 years)	-3.years(-8.4;-0.7)p = 0.024
*Multisystem vs*. *Single system*	13 (76%) vs. 4	6 (60%) vs. 4	16%(-20;53%)p = 0.365
*Reactivating or chronic active LCH*	15 (88%)	6 (60%)	28%(6;62%)p = 0.088
*Risk lesions for ND-LCH*: *Diabetes insipidus / Craniofacial lesions*	11 / 6	6 / 5*	Odds Ratio 1.53(0.32; 7.37)p = 0.591
*Previous chemotherapy ongoing*	15 (88%)7 (41%)	9(90%)4(40%)	-2%(-26; 22%)p = 0.888/1%(-37; 39%)p = 0.952

This is the Table 1 footnote.

* one patient had both risk factors

Finally, 27 patients (16 males, 11 females) were evaluable for the analysis. Their median age at the study entry was 8.7 years (range, 1.4 to 27.5; quartiles: 5.3, 8.7, 14.6 years). LCH had been diagnosed at a median age of 2.9 years (range, 4 months -18 years; quartiles: 1.5, 2.9, 7 years). Seventeen patients had DI, which was already present at the time of the diagnosis of LCH (n = 9) or developed during the course (n = 8). Nineteen of the 27 patients had multisystem, and eight single-system disease. Recurrent or chronic active disease was present in 21 patients (78%), 24 had received chemotherapy, and 11 of them were being treated during the study.

Median age at the study entry was similar in groups 1 and 2 (8.2 vs. 10.2 years; p = 0.670). The duration of the disease (time elapsed between the onset of LCH and the first MRI evaluation), did not differ in the two groups (p = 0.688). Patients in group 1 had a significantly younger age at the onset of LCH than those in group 2 (2 vs. 5.5 years; p = 0.024). Multisystem and reactivating disease appear to be more frequent in patients in group 1, although failing to reach the statistical significance (S1 Table and Table 1).

### Reference standard

#### Neuroimaging (S2 Table *)*

We identified seventeen patients with ND-LCH, ten of whom had no previous evidence of neurodegenerative alterations. All patients exhibited atypical MRI pattern with prevalent involvement of the dentate nucleus and the medium cerebellar peduncles (S1 Fig). In six patients cerebellar abnormalities were very mild (grading:1). Signal abnormalities were also observed in other sites: supra-tentorial white matter (n = 8), brainstem (n = 7), and basal ganglia (n = 1). MRS disclosed an abnormal NAA/Cr ratio in the cerebellum of eight patients of group 1 while was normal in all patients in group 2.

### Diagnostic tests

#### Neurological examination (S2 Table)

Neurological examination was abnormal in 10 patients, nine with (group 1) and one without (group 2) ND-LCH, of which seven were subjectively healthy. The Scale for the Assessment and Rating of Ataxia (S.A.R.A) revealed an abnormal score (range, 1–39) in nine, while only pyramidal signs (hyperreflexia, ankle clonus and Babinski sign) were present in the remaining. Barthel index reached the maximum score (100) in all patients except two (#7,#15), who scored 3 and 80, respectively.

#### Neurophysiological assessment (S2 Table)

Evoked potentials were performed in all 27 patients. In one patient VEPs were unreliable due to technical reasons. SEPs were abnormal in 11 patients of group 1, bilaterally in six. The N20 response was delayed in four (#1,#8–10), of decreased amplitude in four (#2,#4,#5,#16), and absent in three (#7,#14,#15) patients. The P14 response was absent in two patients (#7,#15) and of decreased amplitude in one (#16). The N13 component was absent in one patient (#15). BAEPs were abnormal in five patients of group 1 (#1,#3,#7,#15,#17) and in one of group 2 (#23). VEPs were normal in all patients. EEG was normal in all patients but one (#5), who had fronto-central spikes during sleep but never experienced seizures.

#### Neuropsychological assessment (S2 Table)

Neuropsychological evaluation was normal in all the 18 patients tested, with two exhibiting a discrepancy between verbal and non-verbal performances (#2,#21). Two patients had a specific learning disorder (#10,#19).

### Evaluation of the diagnostic performance of the diagnostic studies

#### Statistical analysis (Table 2, S3 Table and S4 Table)

The best performance for prediction of ND-LCH verified using MRI was observed for SEPs (ROC area = 0.85; 95%CI 0.66–0.96) with high specificity and lower sensitivity (70%), followed by NE (ROC area = 0.71; 0.50–0.86). When using ordinal grading MRI abnormalities, again SEPs had the best discrimination performance (Harrell’s c = 0.80; 0.70–0.90). Defining a cut-off for the ordinal grading MRI abnormalities of 1, SEPs showed 82% specificity and 90% sensitivity.

**Table 2:** Sensitivity and specificity, ROC area, PPV, NPV, number of patients of NE, BAEPs, SEPs, MRS, NPS for diagnostic test of MRI and grading of MRI ND-LCH.95% Confidence Intervals in parentheses.

	Sensitivity	Specificity	ROC area	PPV	NPV	N° patients
**MRI**						
NE	52.9%(27.8%-77.0%)	90.0%(55.5%-99.7%)	0.71(0.50–0.86)	90.0%(55.5%-99.7%)	52.9%(27.8%-77.0%)	27
BAEPs	29.4%(10.3%-56.0%)	90.0%(55.5%-99.7%)	0.60(0.39–0.78)	83.3%(35.9%-99.6%)	42.9%(21.8%-66.0%)	27
SEPs	70.6%(44.0%-89.7%)	100.0%(69.2%-100%)*	0.85(0.66–0.96)	100.0%(73.5%-100%)*	66.7%(38.4%-88.2%)	27
MRS	52.9%(27.8%-77.0%)	90.0%(55.5%-99.7%)	0.71(0.50–0.86)	90.0%(55.5%-99.7%)	52.9%(27.8%-77.0%)	27
NPS#	22.2%(2.8%-60.0%)	71.4%(29.0%-96.3%)	0.47(0.20–0.70)	50.0%(6.8%-93.2%)	41.7%(15.2%-72.3%)	16
**Grading of MRI cut-off grading level > 1**						
NE	80.0%(44.4%-97.5%)	88.2%(63.6%-98.5%)	0.84(0.66–0.96)	80.0%(44.4%-97.5%)	88.2%(63.6%-98.5%)	27
BAEPs	30.0%(6.7%-65.2%)	82.5%(56.6%-96.2%)	0.56(0.35–0.75)	50.0%(11.8%-88.2%)	66.7%(43.0%-85.4%)	27
SEPs	90.0%(55.5%-99.7%)	82.4%(56.6%-96.2%)	0.86(0.66–0.96)	75.0%(42.8%-94.5%)	93.3%(68.1%-99.8%)	27
MRS	50.0%(18.7%-81.3%)	70.6%(44.0%-89.7%)	0.60(0.39–0.78)	50.0%(18.7%-81.3%)	70.6%(44.0%-89.7%)	27
NPS#	14.3%(0.4%-57.9%)	66.7%(29.9%-92.5%)	0.40(0.15–0.65)	25.0%(0.6%-80.6%)	50.0%(21.1%-78.9%)	16

This is Table 2 footnote.

BAEPs: brainstem auditory evoked potentials; MRI: Magnetic Resonance imaging; MRS: Magnetic Resonance Spectroscopy; NE: neurological examination; NPS: neuropsychological evaluation; NPV: negative predictive value;PPV, positive predictive value; ROC: Receiver operating characteristic;SEPs: somatosensory evoked potentials; VEPs: visual evoked potentials.

#: Neuropsychological evaluation was performed in 16 out of 27 patients either for patient’s refusal or because no neuropsychologist was available the date of planned evaluation.

(*) one-sided, 97.5% confidence interval

Adding NE to the diagnostic protocol did not improve significantly the discrimination performance, but there was an improvement in average sensitivity on ordinal grading MRI abnormalities over SEPs alone, and overall the integrated discrimination improved by 7.6% (-6%; +21%). Based on previous findings, we defined a diagnostic protocol including SEPs and NE to assess whether patients with ND-LCH can be predicted as such prior to MRI. Both tests can be negative or positive or only one of the two can be positive. On the basis of these three thresholds, we calculated sensitivity and specificity area under the ROC curve.

### Follow-up studies (Table 3)

We monitored clinical, MRI and neurophysiological changes in patients with ND-LCH. All patients but two (#5 and 7) of group 1 underwent repeated evaluations including MRI, MRS, NE, BAEPs and SEPs. We excluded VEPs and EEG due to their low sensitivity. In patient #7 EPs were not re-evaluated as already severely impaired at the first evaluation; patient #5 was lost from the follow-up.

**Table 3:** Neurological, neurophysiological, radiological assessment of patients in Group 1 at short term follow-up.

*Pt*	*Time interval(months)*	*NE*	*BAEPs*	*SEPs*	*MRI/grading*	*MRS*
1	0	Slight tremor at nose-finger test (SARA:1)	Bil increased I-V interval	L N20 delayed latency	Cerebellum/1	N
	12	Slight tremor at nose-finger test (SARA:1)	Bil increased I-V interval	L N20 delayed latency and abnormal waveform	Cerebellum/1	N
2	0	R deafness, L clonus, mild ataxia (SARA: 2)	N	L N20 abnormal waveform and decreased amplitude	Cerebellum,sWM, brainstem/2	N
	42	Slight tremor at nose-finger test, clonus in the left lower limb, enhanced deep tendon reflexes, R deafness (SARA: 2)	Right increased I-V interval	Bil N20 delayed latency	Cerebellum,sWM, brainstem/2	N
3	0	Slight ataxia (SARA:1)	Bil increased I-V interval	N	Cerebellum,brainstem/3	N
	15	Slight ataxia (SARA:1)	Abnormal R V component waveform	N	Cerebellum,brainstem/3	N
4	0	Clonus and enhanced deep tendon reflexes in the lower limbs (SARA:0)	N	Bil N20 decreased amplitude	Cerebellum,sWM/2	Ab
	14	Slight hemiparesis, clonus and enhanced deep tendon reflexes in the lower limbs (SARA:0)	N	L absent N20	Cerebellum,sWM/2	Ab
5	0	L arm weakness, left dysmetria, nystagmus (SARA: 4)	N	R N20 abnormal waveform and decreased amplitude	Cerebellum,sWM,brainstem/3	Ab
	NA	NA	NA	NA	NA	NA
6	0	N (SARA:0)	N	N	Cerebellum/1	N
	34	Bilateral slight tremor at nose-finger test, clonus in the left lower limb, R enhanced deep tendon reflexes (SARA:1)	N	Right N20 decreased amplitude	Cerebellum/1	N
7	0	Tetraparesis, dysphonia, dysphagia, dysarthria (SARA:39)	IV and V abnormal waveforms on the R side	Bil absent P14 e N20 responses	Cerebellum,sWM,BG, brainstem/4	Ab
	50	Impaired consciousness, worsening of dysarthria and dysphagia	NA	NA	Cerebellum,sWM, BG, brainstem/4	Ab
8	0	N (SARA:0)	N	BilN20 delayed latency	Cerebellum/4	Ab
	17	NA	N	BilN20 delayed latency	Cerebellum/4	Ab
9	0	N (SARA:0)	N	Bil N20 delayed latency	Cerebellum,sWM, brainstem/3	Ab
	33	N (SARA:0)	N	Bil N20 delayed latency (worsened)	Cerebellum,sWM, brainstem/3	Ab
10	0	N	N	Bil delayed N20 latency	Cerebellum,sWM/1	Ab
	24	Slight tremor and dysmetria at nose-finger test (R>L), clonus and enhanced deep tendon reflexes in the lower limbs (R> L) (SARA:1)	N	R N20 decreased amplitude, L absent N20	Cerebellum,sWM/1	Ab
11	0	N (SARA:0)	N	N	Cerebellum/1	N
	22	N (SARA:0)	N	N	Cerebellum/1	Ab
12	0	N (SARA:0)	N	N (N20 at upper limits)	Cerebellum, sWM/2	N
	30	N (SARA:0)	N	N (N20 at upper limits)	Cerebellum, sWM/2	N
13	0	N (SARA:0)	N	N	Cerebellum, sWM/1	Ab
	33	N (SARA:0)	N	N	Cerebellum, sWM/1	N
14	0	R tremor and dysmetria at nose-finger test (SARA:2)	N	R N20 slight reduction	Cerebellum,sWM, BG, Brainstem /2	N
	23	Bil tremor and dysmetria at nose-finger test (SARA:2)	N	R N20 slight reduction	Worsening in sWM and BG, Cerebellum/2	N
15	0	Dysarthria, dysphagia, ataxia, bradykinesia, hypertonus, slight R hemiparesis, dysmetriaatnosefingertest (R>L), clonusandenhanceddeeptendonreflexes (R> L) (SARA:14)	Bil increased I-V interval	Bil absent N13, P14 and N20	Cerebellum, brainstem/4	Ab
	8	Dysarthria, ataxia, bradykinesia, hypertonus, slight R hemiparesis, dysmetriaatnosefingertest (R>L), clonusandenhanceddeeptendonreflexes (R> L). Dysphagiaimprovement (SARA:14)	Bil increased I-V interval (R>L)	Bil absent N20	Cerebellum, brainstem/4	Ab
16	0	Ldysmetria and tremor at nose-finger test, R clonus (SARA:2)	N	L P14 and N20 decreased amplitude	Cerebellum,sWM/2	N
	40	Bildysmetria and tremor at nose-finger test, L dysmetria at the heel-shin slide (SARA:2,5)	Bil increased I-V interval	R absent N20, L N 20 delayed latency	Cerebellum,sWM/2	N
17	0	N (SARA: NA)	Increased I-V interval on the R side	N	Cerebellum/1	Ab
	24	N (SARA: 0)	BilIncreased I-V interval	N	Cerebellum/1	Ab

This is Table 3 footnote.

Ab: abnormal; BAEPs: brainstem auditory evoked potentials; BG: basal ganglia; Bil: bilateral; EEG: electroencephalogram; FSIQ: full scale intelligence quotient; L: left; MRI: Magnetic Resonance imaging; MRS: Magnetic Resonance Spectroscopy; N: normal; NA: not available; NE: neurological examination; NP: not performed; NPS: neuropsycological evaluation; PIQ: performance intelligence quotient; R: right; SARA: Scale for the Assessment and Rating of Ataxia; SEPs: somatosensory evoked potentials; SLD: specific language disorder; sWM: supratentorial white matter; VEPs: visual evoked potentials; VIQ: verbal intelligence quotient.

The median follow-up duration was 24 months (range, 8–50 months). In five patients(#1, 3, 11–13) neurophysiological and neurological findings remained unchanged. A worsening of EP results was observed in eight patients (#2, 4, 6, 8–10, 16,17), five of which (#2, 6, 8–10) also showed neurological worsening.

In patient #14 neurological symptoms worsened without EP impairment. In patient #15 SEPs slightly improved.

MRI remained unchanged in all patients but one (#14) in whom a progression of white matter and globus pallidus abnormalities was observed. MRS findings appeared modified in two (#11, 13) patients.

## Discussion

We applied a multidisciplinary diagnostic protocol for assessing and following-up patients with early-stage ND-LCH. In this study,an extended structural MRI protocol allowed to identify neurodegenerative abnormalities in patients at risk for ND-LCH before neurological impairment and neurophysiological abnormalities became obvious. This result is of relevance as brain MRI is not routinely performed in patients at risk for ND-LCH and targeted MRI protocols to detect neurodegenerative abnormalities have not been developed yet. MRI abnormalities in patients with ND-LCH have been described since 2000 [6, 9]. This same pattern is confirmed in our series, with the cerebellum being the most frequent and earliest site of involvement, sometimes in association to brainstem, basal ganglia and hemispheric white matter. We also confirmed the lack of concordance between the degree of neurological impairment and the topography, extent and grading of the neurodegenerative lesions in most patients [16, 27].

While structural MRI appears to be the reference standard, SEPs is the test with the highest capability to predict ND-LCH and discriminate its grading. Remarkably, SEPs were abnormal also in patients with noneurological signs or who only exhibited subtle deficits. In two previous studies on patients with ND-LCH, which included VEPs, EEG and BAEPs, abnormal EPs were found in up to 40% of patients[15, 19]. In our study, 82% of patients with ND-LCH had some kind of EP abnormality. This discrepancy is related to the use of SEPs, as the remaining evoked potentials modalities were less contributive. VEPs were normal in all patients with ND-LCH; only one patient had mild EEG abnormalities during sleep but never had seizures; although BAEPs turned out to be more sensitive than SEPs in selected cases, their overall predictive value was lower. In contrast to previous observations[19, 21], none of our patients had cognitive impairment or working memory deficits.

We noticed that subtle abnormalities may be difficult to identify in a single patient in the early pre-symptomatic stages. Regarding this issue, the combination of neurological examination, targeted MRI protocols, and SEPs had the highest diagnostic value. MRS might provide interesting correlations between morphology and function. A decreased peak of N-acetyl-aspartate in the cerebellum of patients with ND-LCH was described in single case reports[28, 29]. In our study, spectroscopy showed high sensitivity and specificity in identifying patients with ND-LCH and disclosed abnormalities also in patients with minor or no neurological deficits. In the short term follow-up, structural MRI remained unchanged in all but one patient in whom neurological manifestations had worsened. MRS modifications appeared in two patients, suggesting that MRS may usefully integrate structural MRI.

During follow-up we also identified neurological and neurophysiological changes in most patients with ND-LCH, despite structural brain MRI did not show signs of progression. An integrated neurophysiological and neurological protocol appears necessary to monitor the progression of the disease, also in view of possible new therapeutic approaches. Yet, limited knowledge of the pathogenic mechanism of demyelination, without any available animal model of ND-LCH, has impaired our ability to interpret MRI alteration and their changing over time [5].

Recent studies of large cohorts of patients in the prospective trials LCH I to III have shown that a younger age at diagnosis does not represent an independent, adverse prognostic factor for treatment response and survival[30–32]. In our study, patients with ND-LCH had a significantly younger median age at the onset of LCH than those without neurodegenerative alterations. Since the time elapsed from the onset of LCH to the first MRI evaluation was similar in patients with and in those without ND-LCH, our findings suggest that the risk of developing ND-LCH does not depend on the duration of the disease only. It can be hypothesized that a subset of patients with LCH might be individually prone to develop “early onset LCH” followed by ND-LCH. Whether genetic polymorphisms in still undefined genes may play a role in the development of LCH, and of ND-LCH in particular, remains an open issue[5, 33]. Additionally, the hypothesis that LCH may be induced or at least triggered by a viral infection has been recently emphasized after elevated amounts of Merkel cell polyomavirus DNA were found in the peripheral blood cells of patients with LCH[34]. Whether neurodegeneration may be a late manifestation of the viral pathogen in LCH also remains an open issue[5].

The main unmet medical need in ND-LCH remains treatment. Since reversal of clinical manifestations in patients with overt impairment is unrealistic, hopes should point to an early start of a potentially effective therapy. Our multidisciplinary approach can allow to identify asymptomatic or pauci-symptomatic patients with ND-LCH in order to start treatment early and possibly improve prognosis. A longitudinal therapeutic study is currently ongoing in our center and will hopefully help solve this issue. The possible therapeutic use of target therapy at least in the subset of patients with V600EBRAF mutation should be carefully explored in a scientific setting[35].

Our study has limitations. The number of patients is relatively small; yet, given the rarity of the disease, and especially of ND-LCH, recruiting a larger number of patients would have needed a very long time or a multicenter setting, thus introducing additional potential biases. We also acknowledge that a study limited to prospective observation of newly diagnosed patients with LCH could have helped in describing the natural history of the disease, but this might require an extremely long time interval, which would impair feasibility. A correlation between time of follow-up and the probability to observe ND-LCH has been proposed [16]. To this issue, it is relevant that the duration of the disease was comparable between patients in group 1 and those in group 2, thus confirming that patients at risk but who did not show ND-LCH were not simply observed too early, at least compared to those with ND-LCH.

## Conclusions

We propose a novel protocol of evaluation for patients with LCH, with the main purpose of identifying and monitoring patients with ND-LCH while still in a pre- or pauci-symptomatic stage. Patients with ND-LCH deserve a targeted structural MRI study for early identification of demyelination. Spectroscopy may then usefully complement structural MRI. SEPs are promising in detecting initial brain dysfunction. The combined use of SEPs and careful neurological evaluation may represent a valuable, low-cost, well-tolerated and easily available methodology to monitor patients from pre-symptomatic to symptomatic stages of ND-LCH. The wider use of our multidisciplinary protocol might allow the selection of patients for early therapeutic intervention, hopefully providing the basis for future therapeutic trials.

## Supporting Information

S1 Fig MRI and EP features of patients 4 and 7. (DOCX)

S1 Methods (DOCX)

S1 References (DOCX)

S1 Table Clinical characteristics of the study population. (DOCX)

S2 Table Neurological, neurophysiological, neuropsychological and radiological findings of the 27 patients with LCH divided in two groups according to the presence of either MRI alterations specific for ND-LCH (Group 1) or only of lesions at risk for ND-LCH (Group 2). (DOCX)

S3 Table Harrell’s c, Integrated Discrimination Improvement, average Improvement in Sensitivity and Specificity, 95% confidence intervals, number of patients, of NE, BAEPs, SEPs, MRS, NPS for diagnostic tests of ordinal grading of MRI ND-LCH. p-values of testing the null hypothesis of no contribution of adding NE to SEPs in the diagnostic protocol.
(DOCX)

S4 Table Positive and Negative Likelihood ratios of NE, BAEPs, SEPs, MRS, NPS for diagnostic tests of ND-LCH. (DOCX)
