# Supplementary material for: Early Diagnosis and Monitoring of Neurodegenerative Langerhans Cell Histiocytosis
Source: PLoS One. 2015 Jul 15;10(7):e0131635. doi: 10.1371/journal.pone.0131635 (PMC4503531; doi:10.1371/journal.pone.0131635)
Supplement: S1 Fig — (DOCX) [file pone.0131635.s001.docx]

**S1 Figure: MRI and EP features of patients 4 and 7**


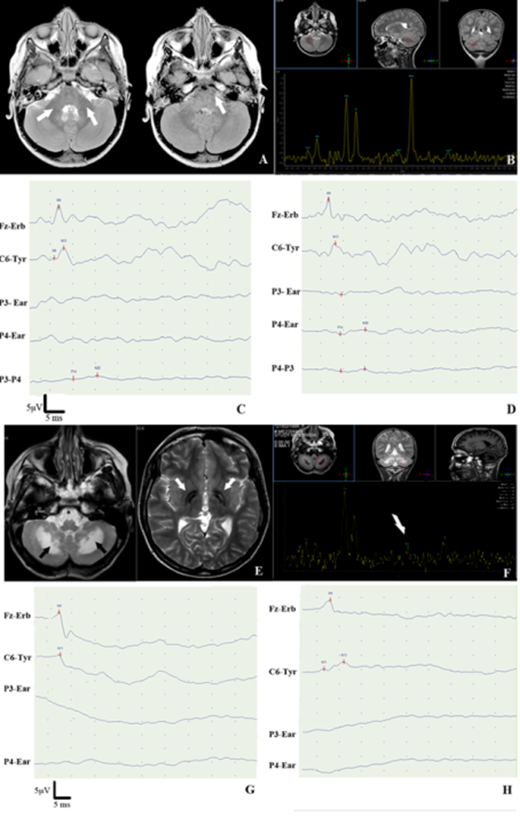


**Legend to S1_Fig.**

**A,B,C and D**: patient 4.

A. T2 weighted and FLAIR MRI images showing cerebellar hyperintensities mainly involving the dentate nuclei (white arrows).B: MRS showing normal values at cerebellar level. C and D: SEPs after (C) right and (D)left median nerve stimulation showing bilateral reduction of N20 potential amplitude.

**E,F,G and H**: patient 7.

E. T2 weighted MRI images showing bilateral extended cerebellar areas of increased signal intensity and symmetric hypointensities in the globuspallidus (white arrows).

F. MRS showing severe decrease of NAA/Cr ratio at cerebellum level (white arrow).

G and H: SEPs after (C) right and (D)left median nerve stimulation showing bilateral absence of the P14 and N20 responses.
