# Supplementary material for: Early Diagnosis and Monitoring of Neurodegenerative Langerhans Cell Histiocytosis
Source: PLoS One. 2015 Jul 15;10(7):e0131635. doi: 10.1371/journal.pone.0131635 (PMC4503531; doi:10.1371/journal.pone.0131635)
Supplement: S1 Methods — (DOCX) [file pone.0131635.s002.docx]

**S1 Methods**

**Neuroimaging**

Patients were scanned for conventional anatomic MRI and Magnetic Resonance Spectroscopy (MRS) 3 T scanners (Philips Achieva, Best, The Netherlands) using a 8-channels head coil. A protocol including axial T2 FSE, FLAIR, DIR and DWI, coronal T2 FSE, sagittal T1 3D FFE and 3D FLAIR, SE T1 sequences on the three planes after contrast injection was applied.

Cerebellar and brainstem neurodegenerative lesions were defined as previously reported (Prayer et al, 2004;Prosch et al, 2008) as poorly defined patchy areas of low signal intensity on T1WI and high signal intensity on FLAIR and T2WI.In the basal ganglia, the abnormalities consisted of hyperintensities on T1-weighted images and variable signal intensities on T2-weighted images. These lesions did not show contrast enhancement.

Cerebellar grading was created to assess the severity of neuroradiological neuro-degeneration. We used a score 1 to 4 to indicate the grade of cerebellar lesion involvement from mild to very severe. The severity of grading was assessed either by the extension of the lesions and their signal intensity.

MRS was performed on three regions of interest (supraventricular white matter, basal ganglia and cerebellar hemisphere). Single-voxel MRS of these regions was acquired via point-resolved spectroscopy (PRESS TE = 288 ms)[1] with a volume of 25 x 20 x 15 mm. The resulting spectra were analyzed using FSL (Oxford, UK; FSL, http://www.fmrib.ox.ac.uk/fsl). Metabolite signals, determined by peak integration included the major proton metabolites: N-acetylaspartate (NAA at 2.02ppm), creatine (Cr at 3.03ppm), choline (Cho at 3.24ppm), lactate (Lac at 1.4ppm). An acquisition was obtained without water suppression to measure tissue water content which was then used to normalize concentration of each metabolite thus accounting for tissue fraction in the voxel. Our primary analysis compares metabolites ratios relative to creatine. N-acetylaspartate to creatine ratio (NAA/Cr) is widely used as a marker of neuronal integrity. Based on normative data collected in our laboratory comparing the average NAA/Cr values of the patient group with those of a group of healthy controls, the cutoff we set to differentiate normal from abnormal MRS values in cerebellum was 1.93 ± 0.24[2].

Two different radiologists (S.S., M.M.) reviewed all the images and defined the presence and the localization of parenchymal lesions, their behaviour after contrast media injection and the grading of cerebellar lesions. They were blinded to the neurological and neurophysiological findings of the study.

**Neurological evaluation**

The neurological evaluation included a complete assessment of muscle strength, sensibility, cranial nerve functioning, tonus, gait, coordination, superficial and deep tendon reflexes and specific scales for ataxia (S.A.R.A: Scale for the Assessment and Rating of Ataxia[3]) and disability (Barthel Index[4]).

**Neurophysiological protocol**

The neurophysiological assessment included evoked potentials (EP) and electroencephalogram (EEG), in one or two sessions, depending on their level of collaboration.

EP recording protocols and response labelling were in agreement with IFNS recommendations (Nuwer et al, 1994; Cruccu et al, 2008). EP normative values were set in the Pediatric Neurophysiology Laboratory of Children’s Hospital Meyer, Florence across different age groups. EPs were considered abnormal in the following cases: a) delayed latency and/or interpeak interval, b) decreased amplitude c) asymmetrical findings over the two sides d) abnormal and not reproducible waveforms[5–8].Two trained neurologists evaluated neurophysiological findings blinded to MRI results.

1) Somatosensory evoked potentials (SEPs) after median nerve (MN) stimulation. SEPs were recorded bilaterally using a 5-channel EP/EMG acquisition unit (Myoquick, Micromed, Mogliano Veneto, Italy). Stimuli (duration 0.3 ms, frequency 1.5 Hz) were delivered through skin electrodes at the wrist; stimulus intensity was adjusted slightly above the motor threshold. The recording electrodes were placed over the supraclavicular fossa (Erb’s point), over the sixth cervical vertebra, and over the parietal scalp regions contralateral and ipsilateral to the stimulated side. The Erb’s point electrode was referred to Fz; the cervical electrode was referred to an electrode located immediately above the thyroid cartilage; parietal scalp electrodes ipsilateral and contralateral to stimulation were referred to the earlobe ipsilateral to the stimulated side. We obtained “difference traces” by subtracting ipsilateral from contralateral parietal leads. The ground electrode was placed over the ipsilateral forearm. The filter bandpass was 1-3000 Hz and the analysis time 50 ms. Two averages of 200 trials each were obtained.

We evaluated amplitudes, latencies, waveforms and scalp distribution of the main SEP components: the Erb’s point potential, the cervical N13, the lemniscal P14 and the cortical N20 responses. Amplitudes and peak latencies were measured on the average of two runs. Amplitudes were measured from baseline. We analysed scalp “difference traces” as described above to distinguish subcortical N18 response from the true ‘N20’ response[8].

2) Brainstem Auditory Evoked Potentials (BAEPs). BAEPs were recorded using a 5 channel EP/EMG acquisition unit (Myoquick, Micromed, Mogliano Veneto, Italy). Stimuli were presented monaurally to each ear, via headphones (level of intensity 80 db sound pressure, frequency 10 hz, acquisition time 15 ms). The recording electrode Fz was referred to both earlobes. The bandpass filter was 160-1.6 khz. Two averages of 800 trials each were obtained to increase the signal to noise ratio. Latencies and waveforms of the first five vertex positive peaks (labelled using roman numerals) were analysed. The intervals between the first and the third and five positive peaks (intervals I-III and I-V) were evaluated too.

3) Visual Evoked Potentials. Pattern-shift VEPs (PVEPs) were obtained using a 5-channel EP/EMG acquisition unit (Medelec Synergy, Tecnomed, Pescara, Italy) and a black-and-white checkerboard displayed on a TV screen, which reversed each second. Each check subtended 60° of the visual field. Recordings were performed after monocular full-field stimulation with the active scalp electrode being OZ, referred to CZ. The ground electrode was placed over the forearm. The filter bandpass was set at 2-100 Hz, the analysis time was 500 ms; 200 responses were averaged. Latencies, amplitudes and waveforms of the N75, P100 and N145 components were evaluated.

4) Video-EEG. All the patients underwent 1-hour video-EEG recording while awake and asleep (Micromed System, Mogliano Veneto, Italia), with intermittent photic stimulation (IPS) and hyperventilation. Twenty scalp electrodes (including Oz) were placed according to the International 10-20 system recommendations with simultaneous polygraphic recordings. Electromyogram (EMG) was recorded using pairs of electrodes applied 3 cm apart over deltoids. Electrocardiography (ECG), electromyogram (EMG) and respirogram were performed using bipolar electrodes and a thoracic belt, respectively. Impedance of all electrodes was kept below 10 kOhm. A bandpass filter of 1 to 70 Hz was applied to the EEG signal; EMG activity was filtered from 53 to 300 Hz, ECG from 5 to 30 Hz and respirogram from 0,16 to 5 Hz. The background activity, the sleep architecture and the presence and topography of paroxysmal abnormalities, if any were analysed.

**Neuropsychological assessment**

Neuropsychological evaluation included the following standard clinical tests to measure intellectual efficiency and identify possible specific cognitive deficits.

- 1. Intellectual ability: WPPSI46 for children < 6 years; WISC-R47 for children > 6 years; WAIS-R for patients > 17 years[9–10]; Raven’s Progressive Matrices[11].
  2. Language: naming: Boston Naming Test (BNT)[12]; verbal fluency: Word Fluency test[13]; Comprehension: BVN[14].
  3. Visuospatial processing: line cancellation and bisection.
  4. Memory span test (backward and forward) semantic and episodic memory evaluation[15]; Rey figure[16], Benton Visual Retention Test[17].
  5. Working memory: audio-verbal working memory test.
  6. Praxis: BVN^12^; Rey-Osterrieth Complex Figure[16]; copy of drawings.
  7. Learning reading: words and nonwords reading (accuracy and rapidity), DDE-2 battery[18]
  8. Learning writing: DDO (writing fluidity)
  9. Visuomotor integration: VMI[19]
