# Supplementary material for: Early Diagnosis and Monitoring of Neurodegenerative Langerhans Cell Histiocytosis
Source: PLoS One. 2015 Jul 15;10(7):e0131635. doi: 10.1371/journal.pone.0131635 (PMC4503531; doi:10.1371/journal.pone.0131635)
Supplement: S1 References — (DOCX) [file pone.0131635.s003.docx]

**S1 References**

1. Bottomley PA, Hardy CJ, Argersinger RE, Allen-Moore G. A review of 1H nuclear magnetic resonance relaxation in pathology: are T1 and T2 diagnostic? *Med Phys* 1987;14:1–7.

2. De Stefano N, Narayanan S, Francis GS, et al. Evidence of Axonal Damage in the Early Stages of Multiple Sclerosis and Its Relevance to Disability. *Arch Neurol* 2001;58:65­–70.

3. Schmitz-Hübsch T, Tezenas du Montcel S, et al. Reliability and validity of the International Cooperative Ataxia Rating Scale: a study in 156 spinocerebellar ataxia patients. *Mov Disord* 2006;21:699–704.

4. Granger CV, Dewis LS, Peters NC, Sherwood CC, Barrett JE. Stroke rehabilitation: analysis of repeated Barthel index measures. *Arch Phys Med Rehabil* 1979;60:14–17.

5. Nuwer MR, Lehmann D, Lopes da Silva F, Matsuoka S, Sutherling W, Vibert JF. IFCN guidelines for topographic and frequency analysis of EEGs and EPs. Report of an IFCN committee. International Federation of Clinical Neurophysiology. *Electroencephalogr Clin Neurophysiol* 1994a;91:1–5.

6. Nuwer MR, Aminoff M, Desmedt J, et al. IFCN recommended standards for short latency somatosensory evoked potentials. Report of an IFCN committee. International Federation of Clinical Neurophysiology. *Electroencephalogr Clin Neurophysiol* 1994b;91:6–11.

7. Nuwer MR, Aminoff M, Goodin D, et al. IFCN recommended standards for brain-stem auditory evoked potentials. Report of an IFCN committee. International Federation of Clinical Neurophysiology. *Electroencephalogr Clin Neurophysiol* 1994c;91:12–17.

8. Cruccu G, Aminoff MJ, Curio G, et al. Recommendations for the clinical use of somatosensory-evoked potentials. *Clin Neurophysiol* 2008;119:1705–19.

9. Wechsler D. Manual for Wechsler Preschool and Primary Scale of Intelligence. San Antonio, TX: The Psychological Corporation, 1967.

10. Wechsler D. Wechsler Intelligence Scale for Children - Revised (WISC-R). The Psychological Corporation, New York; 1974.

11. Raven J. Matrix Test. *Ment. Hlth* 1940; I:10–18.

12. Kaplan E, Goodglass H, Weintraub S. The Boston Naming Test: Experimental edition. Boston: Lea &Febinger, 1978.

13. Korkman M, Kirk U, Kemp SL. NEPSY. A developmental neuropsychological assessment. San Antonio, TX: The Psychological Corporation,1998.

14. Bisiacchi PS, Cendron M, Gugliotta M, Tressoldi PE, Vio C. BVN 5-11 Batteria di valutazione neuropsicologica per l’età evolutiva. Trento: Erickson, 2005.

15. Orsini A, Grossi D, Capitani E, Laiacona M, Papagno C, Vallar G. Verbal and Spatial Immediate Memory Span: Normative Data from 1355 Adults and 1112 Children. *It J Neurol Sci* 1987; 8:539–548.

16. Caffarra P, Vezzadini G, Dieci F, Zonato F, Venneri A. Rey-Osterrieth complex figure: normative values in an Italian population sample. *Neurol Sci* 2002;22:443–447.

17. Benton Visual Retention Test, Revised Edition. New York: Psychological Corporation, 1992.

18. Sartori G, Job R, Tressoldi PE. Batteria per la valutazione della dislessia e della disortografia evolutiva. Firenze: Organizzazioni speciali,1995.

19. Beery KE, Beery NA. Beery VMI with supplemental developmental tests of visual perception and motor coordination administration, scoring and teaching manual (5th Ed.). Minneapolis: NCS Pearson, 2004.
