# Supplementary material for: Early Diagnosis and Monitoring of Neurodegenerative Langerhans Cell Histiocytosis
Source: PLoS One. 2015 Jul 15;10(7):e0131635. doi: 10.1371/journal.pone.0131635 (PMC4503531; doi:10.1371/journal.pone.0131635)
Supplement: S1 Table — (DOCX) [file pone.0131635.s004.docx]

**S1 Table**: **Clinical characteristics of the study population**

|  | ***Pt*** | ***Sex*** | ***Age***  ***at onset*** | ***Age at first***  ***abnormal MRI*** | ***Age at study*** | ***Site of disease*** | ***DI***  *age at onset* | ***CF*** | ***Reactivation***  *Site (number)* | ***Therapy before study*** | ***Therapy at study*** |
| --- | --- | --- | --- | --- | --- | --- | --- | --- | --- | --- | --- |
| **Group 1** | 1 | M | 2y1m | 9y1m | 9y4m | UFB | - | + | - | VBL, PDN | - |
|  | 2 | M | 2y | 5y | 6y6m | MFB, H-P | 4y | + | skull, hip (2) | VBL, PDN, MTX, 6-MP | MTX, 6-MP |
|  | 3 | F | 10y | NA | 11y4m | H-P, UFB | 2y9m | - | lower limb (1) | - | - |
|  | 4 | F | 23m | NA | 5y7m | Skin, MFB, H-P | 5y | + | lower limb, skull (2) | VBL, PDN, 2cda | - |
|  | 5 | M | 6y3m | 8y9m | 9y2m | H-P, MFB | 5y | - | MFB (1) | - | - |
|  | 6 | F | 7y6m | NA | 22y1m | MFB,H-P,lymphnodes | 7y6m | - | lymphnodes (1) | VBL, PDN | - |
|  | 7 | F | 3y3m | 5y9m | 15y1m | MFB | - | + | skull (4) | VBL, PDN | - |
|  | 8 | M | 20m | 7y6m | 8y1m | MFB, skin | - | + | skull (2) | VBL, PDN, MTX | - |
|  | 9 | M | 9m | NA | 5y | MFB, skin, H-P | 2y6m | + | MFB (4) | VBL, PDN, 2cda, VCR, ARA-C, Indometacin | Indometacin |
|  | 10 | F | 2y3m | NA | 3y1m | MFB | 4y6m | + | skull (1) | VBL, PDN | VBL, PDN |
|  | 11 | F | 10m | NA | 6y6m | MFB, skin,H-P | 5y6m | + | skull, skin (1) | VBL, PDN, RT | - |
|  | 12 | M | 18m | NA | 2y6m | MFB | - | + | skull (1) | VBL, PDN, VCR, ARA-C | VBL, PDN |
|  | 13 | F | 11m | NA | 2y4m | Skin, MFB | - | + | - | VBL, PDN, Indometacin | Indometacin |
|  | 14 | F | 18y | NA | 21y1m | H-P, MFB, lung, mucosa | 15y | + | MFB, mucosa, lung (3) | Zoledronic acid, VBL, PDN | VBL, PDN |
|  | 15 | M | 3y2m | 16y2m | 27y5m | MFB, H-P | 6y | + | skull (1) | Etoposide | - |
|  | 16 | M | 10m | 5y2m | 11y8m | Skin, MFB | 2y5m | + | skull (2) | VBL, PDN | - |
|  | 17 | M | 4m | NA | 22m | MFB, lung, skin | - | + | skull (3) | VBL, PDN, ARA-C, VCR, Indometacin, 2cda | 2cda |
|  |  |  |  |  |  |  |  |  |  |  |  |
| **Group 2** | 18 | M | 9y3m |  | 14y3m | H-P, MFB | 6y | + | skull (2) | VBL, PDN | VBL, PDN |
|  | 19 | M | 2y9m |  | 5y7m | UFB | - | + | - | VBL, PDN | - |
|  | 20 | M | 15y |  | 16y5m | H-P, MFB | 14y4m | - | hip (2) | VBL, PDN | - |
|  | 21 | F | 9y6m |  | 11y5m | H-P, UFB | 6y6m | - | lower limb (1) | VBL, PDN | - |
|  | 22 | M | 4y5m |  | 6y | UFB | - | + | - | - | - |
|  | 23 | M | 6y5m |  | 14y10m | H-P, MFB | 5y | - | hip, skull (2) | VBL, PDN, MTX, 6-MP | - |
|  | 24 | F | 13y6m |  | 16y2m | Thyroid, liver, H-P, lung | 11y7m | - | - | VBL, PDN, 2cda, Liver transplant | Tacrolimus |
|  | 25 | F | 2y4m |  | 8y10m | MFB, H-P | 3y | - | skull (1) | VBL, PDN | - |
|  | 26 | M | 18m |  | 3y7m | MFB | - | + | skull, cervical vertebra (2) | VBL, PDN | Indometacin |
|  | 27 | M | 4y3m |  | 4y10m | MFB | - | + | - | VBL, PDN | VBL, PDN |

This is the table 1 footnote.

Sites at onset are in bold type. H-P: hypothalamo-pituitary; MFB: multifocal bone; UFB: unifocal bone; DI: diabetes insipidus; CF: craniofacial lesions; VBL: vinblastine; PDN: prednisone; 6-MP: mercaptopurine; 2cda: cladribine; VCR, vincristine; ARA-C:cytarabine; MTX: methotrexate;NA: not available as no previous MRI was performed.
