# Supplementary material for: Early Diagnosis and Monitoring of Neurodegenerative Langerhans Cell Histiocytosis
Source: PLoS One. 2015 Jul 15;10(7):e0131635. doi: 10.1371/journal.pone.0131635 (PMC4503531; doi:10.1371/journal.pone.0131635)
Supplement: S2 Table — (DOCX) [file pone.0131635.s005.docx]

**S2 Table:** **Neurological, neurophysiological, neuropsychological and radiological findings of the 27 patients with LCH divided in two groups according to the presence of either MRI alterations specific for ND-LCH (Group 1) or only of lesions at risk for ND-LCH (Group 2).**

|  | ***Pt*** | ***NE*** | ***BAEPs*** | ***SEPs*** | ***VEPs*** | ***EEG*** | ***NPS**** | ***MRI findings/grading*** | ***MRS*** |
| --- | --- | --- | --- | --- | --- | --- | --- | --- | --- |
| *Group 1* | 1 | Slight tremor at the nose finger test (SARA:1) | Bil increased I-V interval | L N20 delayed latency | N | N | N | Cerebellum/1 | N |
|  | 2 | R deafness, L clonus, mild ataxia (SARA: 4) | N | L N20 abnormal waveform and decreased amplitude | N | N | FSIQ: N; VIQ < PIQ | Cerebellum,  sWM, brainstem/2 | N |
|  | 3 | Slight ataxia and dysmetria at the nose-finger test (SARA:2) | Bil increased I-V interval | N | N | N | N | Cerebellum,  brainstem/3 | N |
|  | 4 | L arm and leg slight motor impairment, clonus and enhanced deep tendon reflexes in inferior limbs (SARA:0) | N | Bil N20 decreased amplitude | N | N | N | Cerebellum, sWM/2 | N |
|  | 5 | L arm weakness, left dysmetria, nystagmus (SARA: 4) | N | R N20 abnormal waveform and decreased amplitude | N | R FCT spikes | N | Cerebellum,  sWM,  brainstem/3 | Ab |
|  | 6 | N (SARA:0) | N | N | N | N | NP | Cerebellum/1 | N |
|  | 7 | Tetraparesis, dysphonia, dysarthria (SARA:39) | IV and V abnormal waveforms on the R side | Bil absent P14 e N20 responses | N | N | N | Cerebellum,  sWM,BG, brainstem/4 | Ab |
|  | 8 | N (SARA:0) | N | BilN20 delayed latency | N | N | N | Cerebellum/4 | Ab |
|  | 9 | N (SARA:0) | N | Bil N20 delayed latency | NA | N | N | Cerebellum,  sWM, brainstem/3 | Ab |
|  | 10 | N (SARA:0) | N | Bil N20 delayed latency | N | N | FSIQ: N;  SLD | Cerebellum,  sWM/1 | Ab |
|  | 11 | N (SARA:0) | N | N | N | NP | NP | Cerebellum/1 | N |
|  | 12 | N (SARA:0) | N | N (N20 at upper limits) | N | NP | NP | Cerebellum, sWM/2 | Ab |
|  | 13 | N (SARA:0) | N | N | N | N | N | Cerebellum, sWM/1 | Ab |
|  | 14 | R tremor and dysmetria at nose-finger test (SARA:2) | N | R absent N20 response | N | NP | NP | Cerebellum,  sWM, BG, brainstem /2 | N |
|  | 15 | Dysarthria, dysphagia, ataxia, bradykinesia, hypertonus, slight R hemiparesis, dysmetria at nose finger test (R>L), clonus and enhanced deep tendon reflexes (R> L). (SARA:14) | Bil increased I-V interval; IV and V component abnormal waveform on the R side | Bil absent N13, P14 and N20 responses | N | N | NP | Cerebellum, brainstem/4 | Ab |
|  | 16 | Ldysmetria and tremor at nose-finger test, R clonus (SARA:2) | N | L P14 and N20 decreased amplitude | N | NP | NP | Cerebellum,  sWM/2 | N |
|  | 17 | N (SARA: NA) | Increased I-V interval on the R side | N | N | N | NP | Cerebellum/1 | Ab |
|  |  |  |  |  |  |  |  |  |  |
| *Group 2* | 18 | N (SARA:0) | N | N | N | NP | NP | N | N |
|  | 19 | N (SARA:0) | N | N | N | N | FSIQ: N; SLD | N | N |
|  | 20 | N (SARA:0) | N | N | N | NP | NP | N | N |
|  | 21 | N (SARA:0) | N | N | N | N | FSIQ: N; VIQ > PIQ | N | N |
|  | 22 | N (SARA:0) | N | N | N | N | N | N | N |
|  | 23 | Mild postural  Tremor (SARA: 1) | Abnormal L V component waveform | N | N | N | N | N | N |
|  | 24 | N (SARA:0) | N | N | N | N | N | N | N |
|  | 25 | N (SARA:0) | N | N | N | N | N | N | N |
|  | 26 | N (SARA:0) | N | N | N | N | N | N | N |
|  | 27 | N (SARA:0) | N | N | N | N | N | N | NA |

This is S1 Table 2 footnote.

Ab: abnormal; BAEPs: brainstem auditory evoked potentials; BG: basal ganglia; Bil: bilateral; EEG: electroencephalogram; FSIQ: full scale intelligence quotient; L: left; MRI: Magnetic Resonance imaging; MRS: Magnetic Resonance Spectroscopy; N: normal; NA: not available; NE: neurological examination; N: normal; NP: not performed; NPS: neuropsychological evaluation; PIQ: performance intelligence quotient; R: right; SARA: Scale for the Assessment and Rating of Ataxia; SEPs: somatosensory evoked potentials; SLD: specific language disorder; sWM: supratentorial white matter. VEPs: visual evoked potentials;VIQ: verbal intelligence quotient.
