# Supplementary material for: Early Diagnosis and Monitoring of Neurodegenerative Langerhans Cell Histiocytosis
Source: PLoS One. 2015 Jul 15;10(7):e0131635. doi: 10.1371/journal.pone.0131635 (PMC4503531; doi:10.1371/journal.pone.0131635)
Supplement: S3 Table — p-values of testing the null hypothesis of no contribution of adding NE to SEPs in the diagnostic protocol. (DOCX) [file pone.0131635.s006.docx]

**S3 Table**: **Harrell’s c, Integrated Discrimination Improvement, average Improvement in Sensitivity and Specificity, 95% confidence intervals, number of patients, of NE, BAEPs, SEPs, MRS, NPS for diagnostic tests of ordinal grading of MRI ND-LCH. p-values of testing the null hypothesis of no contribution of adding NE to SEPs in the diagnostic protocol**.

| Grading of MRI | | | |
| --- | --- | --- | --- |
|  | Harrell’s c | 95% CI | N |
| NE | 0.73 | 0.60 – 0.87 | 27 |
| BAEPs | 0.56 | 0.43 – 0.69 | 27 |
| SEPs | 0.80 | 0.70 – 0.90 | 27 |
| MRS | 0.66 | 0.52 – 0.80 | 27 |
| NPS | 0.44 | 0.25 - 0.64 | 16 |

| Integrated Discrimination Improvement analysis  of adding NE to SEPs in the diagnostic protocol | | | |
| --- | --- | --- | --- |
|  |  | 95% CI | p-value |
| IDI | +0.0763 | -0.0600; 0.2126 | 0.2726 |
| IPI grading level 0 | -0.0008 | -0.0926; 0.0910 | 0.9843 |
| ISI grading level 1 | +0.0260 | -0.0736; 0.1256 | 0.5319 |
| ISI grading level 2 | +0.0511 | -0.0404; 0.1426 | 0.2416 |
