# Supplementary material for: Early Diagnosis and Monitoring of Neurodegenerative Langerhans Cell Histiocytosis
Source: PLoS One. 2015 Jul 15;10(7):e0131635. doi: 10.1371/journal.pone.0131635 (PMC4503531; doi:10.1371/journal.pone.0131635)
Supplement: S4 Table — (DOCX) [file pone.0131635.s007.docx]

|  | MRI | | Grading of MRI cut-off grading level > 1 | |
| --- | --- | --- | --- | --- |
|  | LR+ | LR- | LR+ | LR- |
| NE | 5.3 | 1.9 | 6.8 | 4.4 |
| BAEPs | 2.9 | 1.3 | 1.7 | 1.2 |
| SEPs | +∞ | 3.4 | 5.1 | 8.2 |
| MRS | 5.3 | 1.9 | 1.7 | 3.4 |
| NPS | 0.8 | 0.8 | 0.4 | 0.8 |

**S4 Table: Positive and Negative Likelihood ratios of NE, BAEPs, SEPs, MRS, NPS for diagnostic tests of ND-LCH.**

Thisis S1. Table 4 footnote.

BAEPs: brainstem auditory evoked potentials; LR: Likelihood ratios; MRS: Magnetic Resonance Spectroscopy; NE: neurological examination; NPS: neuropsychological evaluation; SEPs: somatosensory evoked potentials.
